# Deep anterior lamellar limbo-keratoplasty for bilateral limbal stem cell deficiency with corneal scarring in chemical injury sequelae: Two case reports

**DOI:** 10.1016/j.ijscr.2022.107409

**Published:** 2022-07-14

**Authors:** Neha Jain, Anahita Kate, Sayan Basu

**Affiliations:** a The Cornea Institute, KAR Campus, LV Prasad Eye Institute, Hyderabad, Telangana, India; b The Cornea Institute, KVC Campus, LV Prasad Eye Institute, Vijayawada, India; c Prof. Brien Holden Eye Research Centre (BHERC), LV Prasad Eye Institute, Hyderabad, Telangana, India

**Keywords:** LSCD, limbal stem cell deficiency, LSCT, limbal stem cell transplantation, PK, penetrating keratoplasty, KPro, keratoprosthesis, AS-OCT, anterior segment OCT, PROSE, Prosthetic Replacement of the Ocular Surface Ecosystem, IOP, intra-ocular pressure, PSC, posterior sub-capsular cataract, BCVA, best corrected visual acuity, DALK, deep anterior lamellar keratoplasty, DAL-LK, deep anterior lamellar limbo-keratoplasty, OCT, optical coherence tomography, Limbo-keratoplasty, Deep anterior lamellar limbo-keratoplasty, Limbal stem cell deficiency, Limbal stem cell transplantation, Corneal scar, Ocular burns, Case report

## Abstract

**Introduction and importance:**

This report describes a new technique of deep anterior lamellar limbo-keratoplasty for the management of bilateral limbal stem cell deficiency (LSCD) with corneal scarring.

**Presentation of cases:**

A 45-year-old male presented with chronic sequelae of ocular chemical injury and had bilateral total LSCD with corneal scarring. The visual acuity (VA) in the right eye was counting fingers. A large diameter deep anterior lamellar limbo-keratoplasty (DAL-LK) was carried out and the donor cornea and limbus were sourced from a single tissue. The VA at the last visit, 2.5 years after the surgery was 20/80. A similar presentation was seen in a 31-year-old male with a VA of 20/320 in the right eye. He underwent a DAL-LK and 3 years after the procedure, the VA was 20/60. Both grafts remained clear with no episodes of rejection until the last follow up visit.

**Discussion:**

Limbal stem cell transplantation with keratoplasty or a keratoprosthesis is required to manage bilateral LSCD with stromal scarring. The former necessitates multiple interventions while the latter is associated with several globe threatening complications. DAL-LK was devised to overcome these disadvantages and offers a simple, single staged technique of simultaneously transplanting the corneal stroma with the limbal stem cells. As the host endothelium is preserved, there is no risk of rejection episodes.

**Conclusion:**

DAL-LK can successfully restore stability of the ocular surface and visually rehabilitate cases with bilateral LSCD and stromal scarring. The procedure has stable long-term outcomes with a good safety profile.

## Highlights

• Bilateral limbal stem cell deficiency requires an allogeneic source of stem cells.
• For concurrent corneal stromal scarring, a keratoplasty is also needed.
• Both procedures can be combined as an anterior lamellar limbo-keratoplasty graft.
• Sourcing tissue from one donor decreases the risk of immunological rejection.
• The single-staged procedure is easy and necessitates minimal immunosuppression.

## 1 Introduction

Diseases that result in a bilateral affliction of the limbal stem cells are usually associated with varying degrees of adnexal and corneal comorbidities [1]. Scarring of the corneal stroma is perhaps one of the most common co-existing pathologies and results in significant visual morbidity. There are several therapeutic options that can be adopted for these cases. These include limbal stem cell transplantation (LSCT) with immediate or sequential lamellar or penetrating keratoplasty, keratoprosthesis (KPro) and combined limbo-keratoplasties [2], [3]. LSCT followed by PK requires two surgeries and use of two donor tissues while the use of a KPro necessitates ordering a special prosthesis which may not be readily accessible [2], [4]. Furthermore, the infrastructure required for the post-operative management following KPro implantation may not be easily available as well. Penetrating limbo-keratoplasty is associated with the risk of immunological graft rejection as the host endothelium is replaced by the allogeneic donor endothelium [5], [6], [7]. In lamellar-penetrating keratoplasty, a peripheral lamellar and central full-thickness keratoplasty is performed, making it a technically more difficult surgery [8]. To overcome the disadvantages of these procedures, we describe a new technique of deep anterior lamellar limbo-keratoplasty (DAL-LK) in two patients of chemical injury sequelae with bilateral LSCD and corneal scarring. This report is as per the SCARE-2020 criteria [9].

## 2 Case presentations

### 2.1 Case 1

**Fig. 1:**
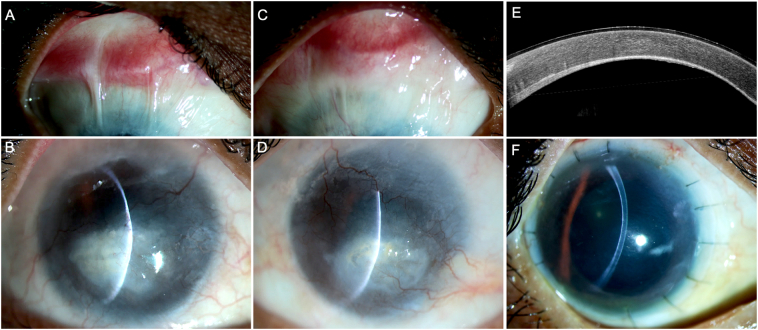
This is a collage of images depicting the clinical presentation of case 1 (A, B) Images of the right eye at presentation with a superior symblepharon, total limbal stem cell deficiency (LSCD), pannus extending from 1 to 5 o'clock and a paracentral leucomatous vascularized scar (C, D) Clinical photograph of the left eye at presentation showing a similar picture with superior symblepharon, total LSCD and a full thickness vascularized scar. (E, F) Image of the right eye 2 years after deep anterior lamellar limbo-keratoplasty with a clear graft, stable ocular surface and a well apposed interface on optical coherence tomography.

A 45-year-old male presented with history of chemical injury with lime in both eyes, 26 years prior to presentation. He complained of diminution of vision in both eyes which had worsened in the 2 years before presenting to us. There was no significant family history. His visual acuity was counting fingers in both eyes. On slit lamp examination, the right eye had a superior symblepharon extending 2 mm into the cornea. A total LSCD with a pannus extending from 1 to 5 o'clock and a 4 × 7 mm leucomatous vascularized scar occupying the inferior half of the cornea was noted (Fig. 1). A similar picture was present in the left eye with a superior symblepharon, total LSCD, a vascularized leucomatous scar as depicted in Fig. 1. B-scan ultrasonography of both eyes did not show any abnormality. He was diagnosed to have bilateral chronic sequelae of chemical injury. A DAL-LK was planned for the right eye to manage the limbal stem cell deficiency (LSCD) and to visually rehabilitate the patient.

The surgery was carried out under local anesthesia by an experienced surgeon. The superior symblepharon was released and the host bed was trephined with a 11.50 mm trephine. A manual lamellar dissection was carried out till the pre-Descemet's layer. A donor corneal button with a death-to-preservation time of 1 h with a healthy limbus and corneal epithelium was chosen and trephined to 12.0 mm to include the limbal cells in the corneal button. It was then sutured to the host bed with 16 interrupted 10-0 nylon sutures. A conjunctival autograft was harvested from the inferior conjunctiva and secured over the bare scleral area with fibrin glue (Tisseel Kit, Baxter AG, Vienna, Austria) to address the superior symblepharon. A lateral para-median permanent tarsorrhaphy was performed with 6–0 polyglactin suture.

On the first post-operative day, vision in the right eye was counting fingers. Mild graft edema was noted with intact sutures. The patient received topical corticosteroids (prednisolone acetate 1 %, 6 times/day) and antibiotics (moxifloxacin 0.5 %, 4 times/day). Systemic immunosuppression was started with oral prednisolone (10 mg once daily) along with oral cyclosporine (50 mg once daily). The patient was followed up monthly for four months and during this time the ocular surface was stable. The graft regained its clarity and the visual acuity improved to 20/125. Topical steroids were tapered to twice a day, oral steroids were decreased to 5 mg/day and oral cyclosporine was continued at the same dose. Two years following the surgery, the patient underwent a cataract surgery for a visual significant PSC. The graft clarity was maintained after the surgery, and this persisted until three months after the surgery at his last follow up visit (Fig. 1). The visual acuity was 20/80 with PROSE scleral contact lenses and the ocular surface was well epithelialized. A normal hyporeflective pattern of corneal epithelium was observed on the OCT with a well apposed graft interface (Fig. 1). The patient was continued on topical corticosteroids (twice daily) along with oral prednisolone (5 mg/day).

### 2.2 Case 2

**Fig. 2:**
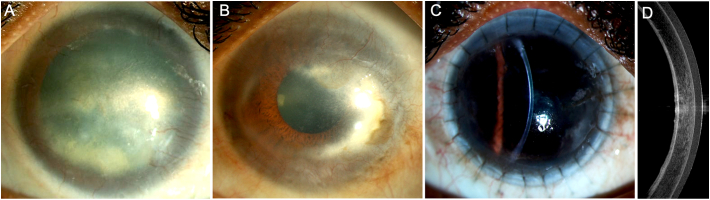
This is a collage of images depicting the clinical presentation of case 2 (A) The right eye has total limbal stem cell deficiency (LSCD) and a nebulo-macular grade scar with vascularization (B) The left eye also has total LSCD with macular grade corneal scarring around the visual axis. (C) A clear graft in the right eye with a well epithelialized surface seen 2 years after the deep anterior lamellar limbo-keratoplasty. A hyporeflective epithelium with a compact stroma is seen on the optical coherence tomography (D).

A 31-year-old male presented with decreased vision in both eyes after having sustained injury with lime 9 years prior to presentation. There was no significant family history. At presentation, his visual acuity in the right and left eye was 20/320 and 20/40 respectively. On slit lamp examination, the right eye had total LSCD and a 6 × 11 mm scar with vascularization (Fig. 2). In the left eye, a 360-degree peripheral conjunctivalization of cornea was seen with macular grade corneal scarring around the visual axis. The posterior segment was normal in both eyes. The patient was diagnosed with both eyes chemical injury sequelae. A DAL-LK was planned in the right eye to address the LSCD and restore clarity to the visual axis.

The surgical procedure was performed using a similar technique as in case 1. The host and donor corneas were trephined with a 11 and 11.25 mm trephine respectively. On the first post-operative day, vision in the right eye was counting fingers. Mild graft edema was noted with a well apposed interface. The patient was started on topical prednisolone acetate 1 % (6 times/day), antibiotics (moxifloxacin 0.5 %, 4 times/day) and oral prednisolone (40 mg/day). The patient also received a short course of oral cyclosporin (50 mg) for 4.5 months after which it was discontinued. The patient had regular follow ups and at the 6-month postoperative visit, the visual acuity had improved to 20/50 and the patient had a well epithelialized clear graft. The patient was continued on topical steroids (once/day) and oral prednisolone (5 mg/day). A visually significant cataract was noted at the 8th month visit for which the patient underwent an uneventful surgery. At the last follow up, three years after the DAL-LK, the visual acuity was 20/60 with a healthy ocular surface and a clear graft (Fig. 2). Similar findings were noted on the OCT which showed a compact graft with a normal hyporeflective corneal epithelium (Fig. 2). A once daily topical steroid dose was maintained.

## 3 Discussion

The most common techniques employed in the management of bilateral LSCD with stromal scarring include LSCT with keratoplasty or a KPro [2], [4]. Although rapid visual recovery is seen following a KPro, the implant is associated with several sight-threatening complications and hence is reserved as a last resort [10], [11], [12]. LSCT with keratoplasty can be performed simultaneously or sequentially. While a single-stage procedure is convenient, it is associated with higher rate of failure of the stem cell transplant [13], [14], [15]. Even with sequentially performed procedure, the corneal graft may yet fail following endothelial rejection. In penetrating limbo-keratoplasty, an eccentrically trephined graft with both the donor cornea and the limbal region is transplanted [5], [7], [16]. These eyes are at a higher risk of immunological rejection of both the donor corneal endothelium and the limbal epithelial stem cells (LESC) [5], [7], [16].

Thus, the current technique was developed to address these issues and provide an efficient modality of managing bilateral LSCD with corneal scarring. By simultaneously transplanting the limbal stem cells and the lamellar cornea, the need for multiple interventions is obviated. This not only decreases the incurred cost for the patient but also eases the logistical issues from a surgical point of view. The procedure is technically simpler and does not require any special training or devices. A large graft was preferred because a smaller graft would require dissection of the same tissue twice. If the limbal tissue is harvested first, it can damage the endothelial cells and vice versa. Also, the astigmatism associated with a small diameter deep anterior lamellar keratoplasty (DALK) is more as compared to a large diameter DALK [17]. Good visual recovery was observed in both the cases in the current report. Additionally, the retention of the host endothelium and sourcing the transplanted tissue from a single donor substantially decreases the risk of a rejection episode. The graft clarity was maintained in both cases over a long follow up period and persisted after the subsequent intraocular procedures. With lesser risk of post-operative complications, a less stringent post-operative follow-up is required.

Systemic immunosuppression was administered in our cases and is recommended because of the close proximity of the graft to the perilimbal vasculature. The underlying ocular diseases can increase the risk of rejection by inducing surface inflammation and corneal vascularization. The viability of the limbal stem cells from the harvested donor tissue is a concern and studies have shown that with increasing death to preservation time there is a progressive decrease in the ability of the LESC to retain their "stemness" or proliferative capacity [18], [19]. Interestingly, the stem cells lose their capacity to reestablish a stable epithelial surface as early as 4 days of preservation despite possessing the stem cell markers [18]. Thus, the utilization of a graft which has been obtained within 48 h is recommended. Finally, the simultaneous use of both the LESC and the lamellar graft from the same donor cornea promotes optimal usage of the precious donor tissue.

## 4 Conclusion

The technique of DAL-LK is a single-staged technique which effectively reestablishes a stable ocular surface and visually rehabilitates patients with bilateral LSCD and corneal stromal opacification. The procedure is technically easier to perform by a trained corneal surgeon and does not rely on special infrastructure. It also has lesser risk of post-operative complications with stable long-term outcomes.

## Consent

Written informed consent was obtained from the patient for publication of this case report and accompanying images. A copy of the written consent is available for review by the Editor-in-Chief of this journal on request.

## Funding

10.13039/501100005809Hyderabad Eye Research Foundation (HERF), Hyderabad, Telangana, India.

## Provenance and peer review

Not commissioned, externally peer-reviewed.

## Ethical approval

Ethics committee approval was not required for this manuscript because it is a clinical case report.

## Author contribution

Study concept or design: SB.

Writing and revising the paper: NJ, AK, SB.

## Research registration

Not applicable.

## Patient perspective

Patient 1: The corneal scar in my eye was unsightly and visually disabling. I am grateful that the current has not only restored my sight but also improved the cosmesis. Patient 2: I had consulted at multiple places and was always explained that I will require multiple surgeries before I can be visually rehabilitated. I was pleasantly surprised and grateful that with one surgery I have had such good results.

## Guarantor

Dr. Sayan Basu.

## Declaration of competing interest

The authors have no conflicts of interest to declare.
